# Controlling nanocage assembly, towards developing a one-health “plug & play” platform for targeted therapy

**DOI:** 10.1039/d5cc03592a

**Published:** 2025-08-08

**Authors:** Yujie Sheng, Mark J. Sutton, Kourosh H. Ebrahimi

**Affiliations:** a Institute of Pharmaceutical Science, King's College London London UK Kourosh.ebrahimi@kcl.ac.uk; b UK Health Security Agency UK

## Abstract

There is a growing interest in developing one-health “plug and play” platforms for making different therapeutics/prophylactics to target various biological entities such as viruses, cancer cells, or bacteria. Such a platform could benefit from advances in artificial intelligence (AI) for sustainable manufacturing. In this respect, naturally occurring protein nanocages, such as iron-storage protein ferritin, are emerging as ideal candidates for various applications in nanomedicine. However, the spontaneous self-assembly of these naturally occurring nanocages has been a bottleneck for their application as a one-health “plug and play” platform. In this review, we will take a fresh look at the application of natural protein nanocages in nanomedicine by discussing our current understanding of their self-assembly process. We highlight our recent progress in engineering ferritin subunits to create a one-health “plug and play” platform technology to develop various therapeutic or prophylactic nanomedicines. We will discuss the advantages of this technology, its implications for understanding nanocage assembly, and potential future application areas.

## Abstract 2

We highlight our recent engineering biology approach in creating a one-health ‘plug and play’ nanocage assembly platform for developing multiple therapeutics to target various entities, including viruses, cancer, and bacteria.

## Graphical abstract

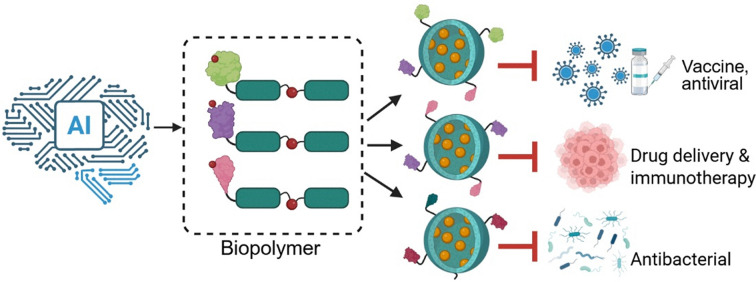

## 1. Introduction

**Fig. 1:**
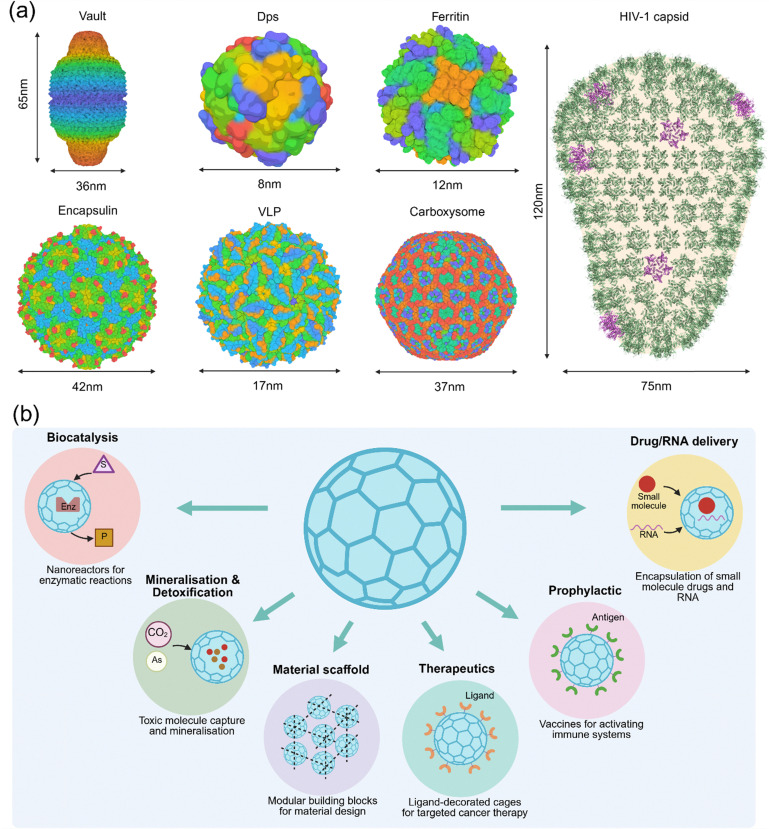
Natural protein nanocages and their applications. (a) The 3-dimensional (3D) structures of common protein nanocages, vault (PDB code: 4V60), Dps (PDB Code: 6LKP), ferritin (PDB Code: 2X17), encapsulin (PDB Code: 6NJ8), VLP (PDB Code: 1CWP), carboxysome minishell (PDB code: 9F0H), and HIV-1 capsid. The structure of the capsid was created using a commercial version of Biorender based on an image from the PDB website (PDB-101: Molecule of the Month: HIV capsid) and structures described in ref. 43 and deposited in PDB. (b) Various applications of natural protein nanocages.

Protein nanocages/microcages are widespread in biology, with various architectures comprising multiple subunits. Examples of natural protein nanocages/microcages include vault,^1,2^ DNA-binding protein from starved cells (Dps) family of proteins,^3^ ferritin,^4^ bacterioferritin,^5,6^ encapsulin,^7^ viral capsids,^8,9^ and bacterial microcompartments (microcages) such as carboxysome (Fig. 1a).^10,11^ These protein cages are made of multiple copies of a single subunit or different types of subunits. The protein shell tightly protects the internal cavity with pores of various sizes and properties, allowing the transportation of specific molecules or ions between the solution and the nanocages’ internal cavity. Consequently, the primary role of most of these protein nanocages and microcages is to perform sensitive biochemical reactions, store toxic molecules, or safely encapsulate a cargo, including genetic material for delivery to a specific target. Among these nanocages, the internal cavity of the vault is exposed to solvent through wide channels, and the cellular function of this fascinating protein complex is still unclear.^1^ As a safe and biocompatible material, protein nanocages' applications in chemistry and medicine has garnered worldwide interest. For example, they have found interest as nanoreactors in biocatalysis,^12–17^ mineralisation of metallic core and removal of toxic molecules such as arsenate and CO_2_,^18–20^ encapsulation of pharmaceutically active molecules such as small-molecule drugs,^21–23^ imaging ligands,^24^ and RNA,^25,26^ a platform to reconstruct 3D materials,^27,28^ and platforms to create vaccines (Fig. 1b).^29–34^ A key step for their application in various areas is controlling the assembly of nanocages. It is required to allow access to the nanocage's internal cavity and to encapsulate various cargoes. It could enable functionalisation of the nanocage surface with multiple ligands. For example, different antigens could be added to the surface of each subunit, and subunits could be mixed to create multivalent vaccines. However, current technologies for controlling assembly require harsh conditions, such as very low pH or high temperatures, and therefore have limited applicability. For example, the widely used method of pH-mediated assembly or disassembly for encapsulating a cargo in various protein nanocages is limited to drugs that are stable under harsh pH conditions. These methods of controlling assembly require multistep processes to encapsulate cargos inside a nanocage and functionalise the nanocage surface with pH-sensitive targeting or therapeutic ligands, such as antibodies or nanobodies. Consequently, bioinspired or synthetic methods are being developed to control the assembly process of nanomaterials under benign conditions. Examples of these methods include novel full-active pharmaceutical ingredient nanodrug (FAND) platforms being developed using small-molecule building blocks,^35–38^ signal-mediated nanocage assembly, such as protease-induced nanocage (PINC) technology, for controlling the self-assembly of natural protein nanocages like ferritin, and unlocking their applications in various areas,^39^ and AI-based design and construction of synthetic protein nanocages.^40–42^

In this review, we will focus on natural protein nanocages since these proteins generally offer a safe and biocompatible platform for biomedical applications. We will first discuss the link between nanocage assembly and biological function and describe current knowledge of the assembly mechanism. Unlike many previous reviews focusing on applying protein nanocages such as ferritin or encapsulin,^30,31,44–47^ we first highlight two different mechanisms of nanocage self-assembly: spontaneous self-assembly *versus* signal-mediated assembly. We discuss how signal-mediated assembly can be used to engineer nanocages and control their self-assembly for biomedical applications, highlighting a “plug & play” technology we have developed, named the protease-induced nanocage (PINC) technology.^39^ We discuss the future direction in the field and highlight the potential of PINC technology to apply AI and create a one-health “plug and play” platform for sustainable manufacturing and rapid development of novel multifunctional therapeutics to target different biological entities such as viruses, bacteria, and cancer cells.

## 2. Spontaneous self-assembly

The spontaneous assembly of nanocages refers to the process whereby subunits come together after expression in a host organism to form the natural nanocages. Hydrophobic patches, hydrogen bonds, and salt-bridge interactions at the interface of subunits drive this spontaneous self-assembly process.^48,49^ The resulting nanocages play fundamental biological functions such as encapsulation of highly reactive metal ions, such as Fe(ii) and Fe(iii), or entrapment of gases, such as CO_2_, as a substrate for biocatalytic transformations. Widely studied examples of these nanocages in order of size include the Dps (DNA-binding protein from starved cells) family of proteins, ferritin, viral capsids, encapsulin, and bacterial microcompartments. In this section, we provide a brief overview of each nanocage, its biological function, and our current understanding of its mechanism of spontaneous self-assembly.

### 2.1. Dps

**Fig. 2:**
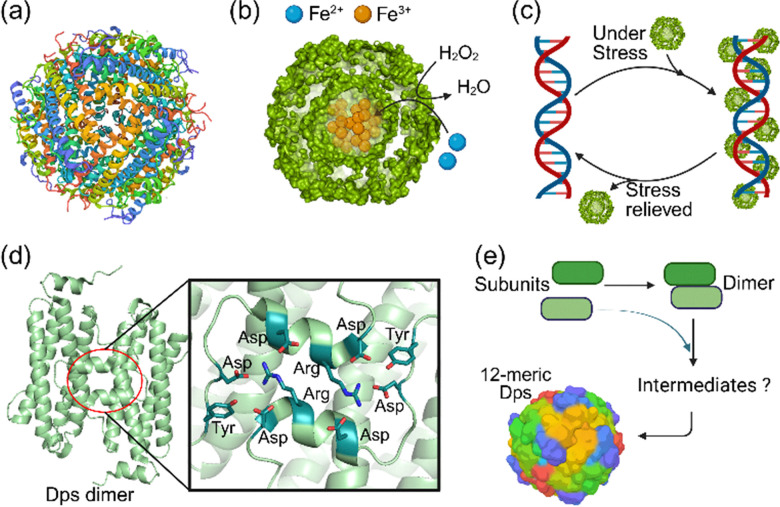
Dps structure, function, and spontaneous assembly mechanism. (a) The 12-meric nanocage structure of Dps (PDB Code: 6LKP). (b) The proposed function of Dps in detoxifying Fe(ii) and H_2_O_2_, and (c) the DNA-binding function of Dps shields DNA against oxidative damage. (d) The crystal structure of the interaction at the interface of two Dps subunits, highlighting key amino acid residues involved in protein–protein interaction. (e) The mechanism of spontaneous self-assembly through dimers. The intermediates are unknown.

Members of the DNA-binding protein from the starved cells (Dps) family of proteins are spherical-shaped nanocages with 3–2 symmetry consisting of 12 identical subunits (Fig. 2a).^3,50^ They are widely present in bacteria, and their nanocage structures protect bacterial cells against oxidative damage, which is key to the virulence of several pathogenic bacteria. This function of nanocages is proposed to be achieved *via* two distinct mechanisms (Fig. 2b).^51^ (i) Binding of ferrous ions (Fe^2+^) to the di-iron catalytic site called ferroxidase centre (FC) and their subsequent reaction with reactive oxygen species like H_2_O_2_ in a controlled fashion. This process prevents the formation of toxic hydroxyl radicals *via* the Fenton reaction. It allows the storage of the insoluble and toxic ferric ion (Fe^3+^) inside the nanocage, which has an internal cavity with a diameter of 4 nm. (ii) Binding to DNA and shielding it from reactive oxygen species (ROS). The self-assembly process appears to be crucial for DNA binding and protection, and its mechanism remains poorly understood. The C-terminal tail of some Dps proteins is shown to be required for nanocage assembly.^52^ The dimers appear to be the most stable species at low pH or salt concentration.^53,54^

Therefore, the self-assembly of Dps nanocage appears to occur *via* the formation of dimers mediated by the C-terminal tail (Fig. 2c).

### 2.2. Ferritin

**Fig. 3:**
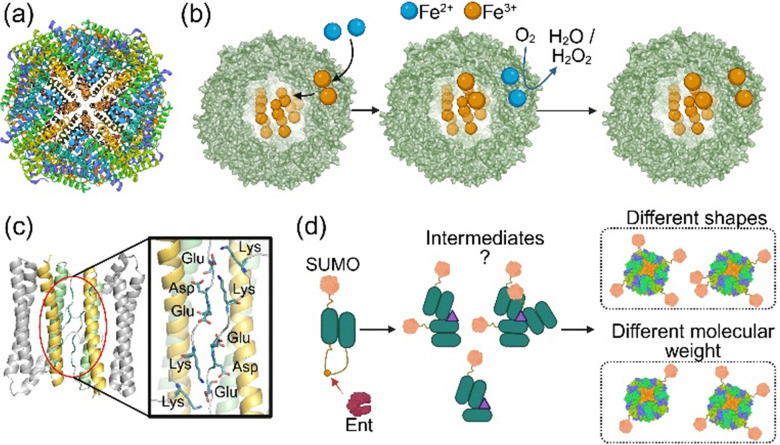
Ferritin structure, function, and self-assembly. (a) The 3D structure of ferritin (PDB code: 2X17). (b) The mechanism of Fe(ii) oxidation and storage. The mechanism demonstrates displacement of Fe(iii) in the ferroxidase centre by incoming Fe(ii) and subsequent Fe(iii) storage and Fe(ii) oxidation. (c) The structure of ferritin dimers. (d) The spontaneous self-assembly starts with the formation of dimers. The intermediates generated during self-assembly are unknown, and the spontaneous assembly after dimer formation is proposed to be stochastic.

The highly conserved protein ferritin is a spherical nanocage composed of 24 subunits with a 4–3–2 symmetry (Fig. 3a).^55–57^ The nanocage's ubiquitous function is to store iron in a bioavailable and non-toxic form.^58^ Experimentally, a maximum of 2000–3500 Fe(iii) atoms per 24-meric shell are stored inside the nanocage.^58^

Each catalytically active ferritin subunit has a ferroxidase centre in the middle. The ferroxidase site catalyses the oxidation of ferrous ions.^55–57^ The ferric ions stay in the ferroxidase centre, are displaced by incoming ferrous ions, and are subsequently stored (Fig. 3b).^59,60^ The self-assembly is shown to be essential for Fe(ii) binding, oxidation, and storage of the resulting Fe(iii) ions as a mineral core.^61^ The first intermediate in nanocage self-assembly is the formation of dimers due to the anti-parallel interaction of two subunits at their interface (Fig. 3c). The evidence supporting dimer formation as the initial step in the self-assembly process was obtained from protein engineering studies, which utilised site-directed mutagenesis to introduce Cu(ii) binding sites at the interface of subunits.^62^ Extensive mutagenesis of the ferritin subunits interface was used to create stable subunits with two Cu(ii) coordination sites at the interface of two subunits. Upon the addition of Cu(ii), monomers formed dimers. These dimers spontaneously generated the 24-meric nanocages. The formation of dimers as the first intermediate in self-assembly was further confirmed by time-resolved small-angle X-ray scattering,^63^ the stochastic nature of the human heteropolymer ferritin self-assembly process,^64^ and linkage of two ferritin subunits using a fusion peptide.^39^ However, the details of the spontaneous self-assembly mechanism after dimer formation have remained elusive. It is unclear how different dimers assemble to generate 24-meric nanocages. The random nature of dimer assembly was proposed by creating precursors to nanocages. We linked a SUMO-protein to the N-terminus of a ferritin subunit fused to a second subunit *via* a linker peptide consisting of an enterokinase cleavage site.^39^

After adding enterokinase and generating ferritin subunits with or without an N-terminal SUMO, the subunits spontaneously self-assembled into nanocages of various sizes and shapes, appearing as a smeared band on native-PAGE. This observation suggests that the self-assembly of dimers occurs *via* multiple pathways, resulting in the assembly of nanocages with varying numbers of SUMO proteins on the surface and/or different shapes (Fig. 3d).

### 2.3. Encapsulin

**Fig. 4:**
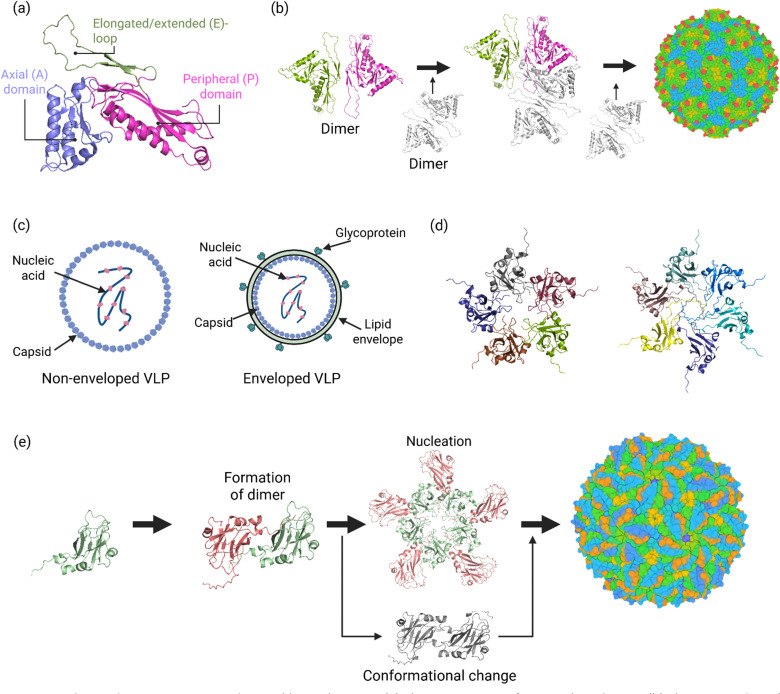
Encapsulins and VLPs structures and assembly mechanisms. (a) The 3D structure of encapsulin subunits. (b) The proposed mechanism of encapsulin self-assembly. It involves the formation of dimers and their subsequent self-assembly. (c) Two different types of VLPs, envelope and non-envelope. (d) The structure of the pentameric and the hexameric units of VLP (PDB Code: 1CWP). (e) The proposed mechanism of spontaneous self-assembly of VLP. The mechanism involves the formation of dimers and subsequently pentameric structures as nucleation sites. These nucleation sites are used to derive the nanocage assembly.

These nanocage compartments were initially identified as high-molecular-weight protein aggregates. The crystallographic analysis revealed that these protein aggregates were indeed nanocages encapsulating functional proteins, and therefore, they were named encapsulins.^65^ These nanocages are widely spread in archaea and bacteria.^66^ They are currently divided into four families depending on the cargo protein, structure, and function.^67^ Family 1 has an encapsulated enzyme, and its function is associated with protecting against oxidative damage caused by reactive oxygen or nitrogen species. The function of family 2 appears to be rescuing sulfur during starvation. Families 3 and 4 are less characterised. Family 3 is associated with the biosynthesis of peptide-based natural products, with the function of the cargo protein being unclear. The function of Family 4 remains a subject of speculation.^7^ The encapsulin subunits have a standard domain structure consisting of an axial (A)-domain, a peripheral (P)-domain, and an elongated/extended (E)-loop (Fig. 4a). The self-assembly process, at least in families 1 and 2, is important for biomineralisation. The self-assembly of encapsulin was studied using native mass spectrometry, suggesting a two-step process.^68^ In the first step, dimers form due to protein–protein interactions between the E-loops of two subunits. These dimers are then repeatedly assembled to generate the spherical-shaped nanocage (Fig. 4b). It is assumed that the cargo protein is encapsulated at some point during the second step. Structural analysis of encapsulin has revealed the presence of specific pores at 5-, 3-, and 2-fold symmetry axes.^69,70^

### 2.4. Viral capsids

Many viral capsids, including phage capsids, spontaneously self-assemble from multiple subunits of a single or different proteins. This process varies depending on the structure, number of subunits, and virus type, and differs between non-enveloped and enveloped viral particles. Viral capsids devoid of any viral material, like DNA or RNA, and viral non-structural proteins, known as virus-like particles (VLPs), are nanocarriers self-assembled from one or more components with a structure like a native virus. These immunogenic nanocages can be broadly divided into two groups (Fig. 4c): non-enveloped and enveloped VLPs.^32^ Non-enveloped VLPs are the result of the self-assembly of viral structural proteins. They are smaller, and their self-assembly depends only on protein–protein interactions between subunits. In contrast, the self-assembly of enveloped VLPs is complex because their structures consist of viral-derived proteins and host self-lipids. Their self-assembly process occurs similarly to that of a viral capsid. The structure and assembly of viral and phage capsids can vary,^71–76^ and the discussion of all these variations is beyond the scope of this review. In almost all cases, the self-assembly generates nanocages that can encapsulate viral genomic material and target specific receptors on the surface of host cells. Here, we briefly discuss the self-assembly of a model of non-enveloped capsid extensively studied, namely the empty capsid from cowpea chlorotic mottle virus (CCMV).^77^ This nanocage has an icosahedral quaternary structure with a diameter of roughly 26–28 nm. It comprises 160 copies of the capsid protein (CP).^78^ These subunits generate 12 pentameric blocks and 20 hexameric blocks in the nanocage structure, in which some subunits are involved in both pentamer and hexamer formation (Fig. 4d). The self-assembly of this CCMV capsid is proposed to occur through a nucleation-and-growth mechanism, enabling the encapsulation of viral genomic material (Fig. 4e).^75^ In this mechanism, five dimeric units are spontaneously self-assembled to create the nucleation core consisting of one pentameric block and five additional CP. This nucleation core is subject to further growth and formation of the nanocage. The protein–protein interactions between CP units and metal ions (Ca(ii)) and RNA binding sites stabilise the nanocage structure. Based on this proposed mechanism, CP dimer formation is the first step in the self-assembly process, similar to the mechanism described for ferritin and encapsulin.

### 2.5. Microcompartments

**Fig. 5:**
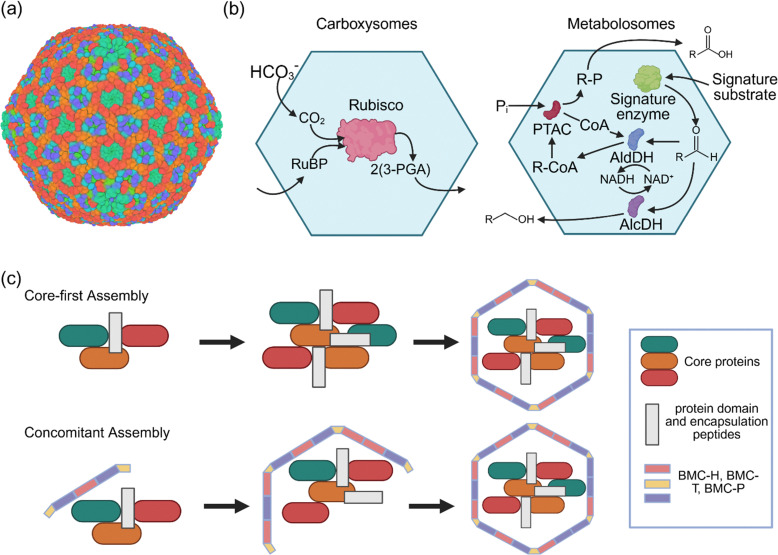
Microcompartments and nanosized bacterial compartments. (a) 3D structure of carboxysome minishell (PDB code: 9F0H). (b) Two known bacterial microcompartments, namely carboxysomes (right) and metabolosomes (left). (c) The mechanism of self-assembly of microcompartments is proposed to occur through two distinct pathways: core-first assembly, whereby different enzymes and proteins are assembled to generate the functional core, and subsequently, the protein shell is formed around the core. Concomitant assembly: whereby the core and the shell assembly occur together.

Bacterial microcompartments (BMCs) have a size roughly in the range of 40–600 nm (Fig. 5a).^79^ These nanosized microbial compartments are lipid-less functional analogues of eukaryotic organelles. They are divided into two groups based on their function (Fig. 5b): carboxysomes and metabolosomes.^80^ Carboxyxomes are nanocage protein organelles responsible for CO_2_ fixation. They encapsulate Rubisco and trap CO_2_ derived from HCO_3_^−^, facilitating its fixation through the Calvin–Benson–Bassham cycle.

Metabolosomes, on the other hand, have diverse initial substrates. They involve a core metabolic reaction that utilises a signature enzyme to generate an aldehyde. Three protein building blocks, named BMC-H (hexamer), BMC-T (trimer), and BMC-P (pentamer), make the shells of these microcompartments.^81,82^ As the name of these building blocks suggests, BMC-H, BMC-T, and BMC-P subunits form hexamers, trimers and pentamers, respectively. The trimers of BMC-T resemble the BMC-H hexamer in size and shape, forming pseudohexamers. Their self-assembly generates a nanocage to prevent the escape of CO_2_ gas or volatile intermediates. The central symmetry axes of BMC-H hexamers and BMC-T pseudohexamers feature pores that connect the internal cavity to the outside environment, allowing for the selective passage of small molecules and metabolites.^81–83^ The self-assembly of BMCs is proposed to proceed *via* two different mechanisms (Fig. 5c): core-first assembly or concomitant assembly. In the proposed core-first assembly mechanism, first, the core proteins aggregate through protein domain interactions or encapsulation peptides.^84,85^ Subsequently, the protein shell is formed, encapsulating the core. In the concomitant assembly process, the core and shell co-assemble simultaneously.^86^

## 3. Protease-cleavage nanocage assembly

In the previous section, we described the spontaneous self-assembly of several proteins. In contrast to spontaneous self-assembly, signal-mediated nanocage assembly requires a biological signal, such as the activity of a protease and/or RNA-binding. In signal-mediated assembly, the protein–protein interactions at the interface between individual subunits cannot occur in the absence of the signal. The presence of a signal, such as a protease, induces conformational changes that are required for protein–protein interactions to occur and for the subsequent assembly of the nanocages. This process is widely used in nature by several viruses, including members of the retroviruses.^87^ Here, we provide a brief overview of studies on signal-mediated HIV-1 assembly and capsid formation as a model for the development of triggered nanocage assembly in other systems.

### 3.1. HIV-1 assembly

**Fig. 6:**
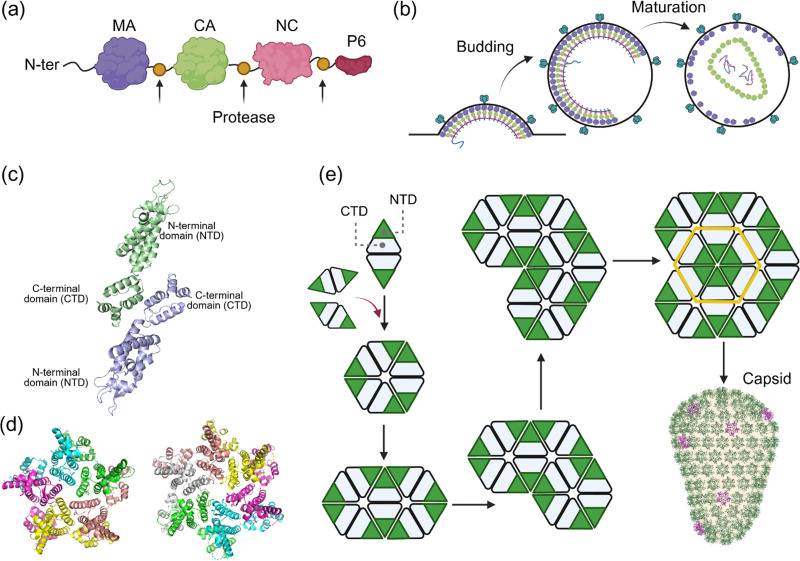
Protease-cleavage nanocage assembly. (a) A cartoon depicting different units of Gag precursors. (b) The protease-cleavage assembly of HIV-1 capsid and maturation. (c) The structure of the CA dimer highlights the interactions between the two C-terminal domains (CTDs). (d) The structure of the pentamer (PDB Code: 3P05) and the hexamer (PDB Code: 3H4E) of HIV-1 capsid. (e) The protease-cleavage induced mechanism of self-assembly of the HIV-1 capsid. The mechanism is proposed to proceed through the spontaneous assembly of dimeric CA units. The structure of the capsid was created using a commercial version of Biorender based on an image from the PDB website (PDB-101: Molecule of the Month: HIV Capsid).

The viral replication and assembly of most Orterviruses, such as HIV-1, includes expression of group-specific antigen, commonly known as Gag. It is a polyprotein molecular assembly machinery comprising all the structural units required for viral assembly and maturation. The synthesis of Gag precursors (Fig. 6a) in the cytosol initiates the process of HIV-1 assembly and maturation, which involves the formation of its capsid nanocage encompassing two copies of viral genomic material.^88^

The Gag precursor should travel to the site of virus assembly at the plasma membrane, where the viral envelope glycoprotein is accumulated, and at the same time, ensure the packaging of two viral RNA copies. To coordinate this complex assembly process, the Gag precursor molecular assembly machinery comprises four protein domains: matrix (MA), capsid (CA), nucleocapsid (NC), and p6 (Fig. 6a), as well as two spacer peptides, SP1 and SP2. The MA domain is essential in targeting the Gag precursors to the inner leaflet of the plasma membrane, the primary site of viral particle assembly. This targeting is achieved through the interaction of the MA domain's highly basic region with the phosphoinositide phosphatidylinositol-4,5-bisphosphate (PtdIns(4,5)P2).^89^ The NC domain of Gag precursors is primarily responsible for binding to viral RNA and packaging.^90^ This domain contains two Cys-Cys-His-Cys zinc-finger-like motifs.^91^ Highly basic residues link these two Zn(ii) binding motifs to increase the affinity for viral RNA. The NC recognises a packing signal in viral RNA,^92^ commonly known as the *ψ*-element, thereby recognising the RNA dimer.^93^ The bulk of evidence suggests that the interaction of the NC domain of a few Gag precursors with RNA dimer occurs in the cytosol, which then localises to the inner leaflet of the cytoplasmic membrane.^88^ These initial Gag–RNA complexes act as a nucleation site, attracting additional Gag precursors and a few GagPol precursors. The CA domain plays a vital role in the nucleation process and formation of multimeric Gag precursors. During nucleation, the viral envelope glycoprotein is incorporated *via* a mechanism that remains to be fully understood. Following the formation of the immature Gag lattice, the nascent viral particle is formed *via* a membrane fission process mediated by cellular ESCRT (endosomal sorting complexes required for transport) machinery.^88,94^ Subsequently, the viral protease-mediated cleavage of GagPol and Gag releases the CA domains, which spontaneously self-assemble to form a conical-shaped capsid, thereby creating the mature virion particle (Fig. 6b). The exact mechanism of self-assembly by which CA forms the capsid is unknown. The interactions between the C-terminal domains (CTDs) of two CA form dimeric units (Fig. 6c). In the mature capsid, the interactions between the N-terminal domains (NTDs) of six CA generate hexamers and pentamers (Fig. 6d). Computational studies suggest a mechanism starting with dimeric CA (Fig. 6e).^76^ In this model, the first three CA dimers generate a nucleation site. The CA dimers are added continuously to generate a central hexamer made by six NTDs, and eventually, the capsid is formed (Fig. 6e). Hence, it appears that the mechanism of signal-mediated assembly by HIV-1 capsid initiates *via* dimer formation, similar to the spontaneous assembly of protein nanocages such as ferritin.

## 4. Nanocage assembly & applications in medicine

Protein nanocages, such as ferritin, are gaining growing interest in biomedical applications.^45^ As a drug delivery vehicle, their uniform and narrow size distribution, along with biocompatibility, offer advantages over synthetic nano-carriers, such as liposomes or polymeric nanoparticles. Their symmetrical structures provide an external surface for functionalisation to develop therapeutics such as vaccines. The possibility of combining delivery and targeting or antigen presentation through surface modification is an attractive feature of such systems. In the case of ferritin, the presence of the N-terminus of the protein at the 3-fold symmetry axis offers the opportunity to use genetic engineering approaches and fuse a monomeric viral surface glycoprotein to generate native-like trimeric viral surface glycoproteins, such as HIV-1 envelope glycoprotein^95^ or SARS-CoV-2 spike glycoprotein.^96^ These virus-like particles are excellent candidates for developing the next generation of efficacious vaccines. mRNA is used to encode the ferritin-subunit vaccine, generating nanocages that present the immunogen inside the body.^97^ One of the grand challenges in advancing ferritin applications as a drug delivery system or platform for making therapeutics, such as mosaic vaccines, has been to control assembly under mild conditions, thereby accessing the internal cavity of nanocages and single subunits. In this section, we first highlight some widely used methods and their use in various therapeutic application areas. We will then highlight and discuss a new “plug & play” technology we have developed, named Protease-Induced Nanocage (PINC) technology,^39^ which enables concomitant drug encapsulation and surface functionalisation under benign conditions.

### 4.1. Chemical and physical disassembly/reassembly

One of the earliest approaches to controlling assembly was based on the disassembly of nanocages under harsh physical or chemical conditions, followed by subsequent reassembly by removing the chemical or physical force, such as pH, temperature, pressure, salt concentration, and chemical denaturation (Table 1).

**Table 1:** A summary of chemical methods used to encapsulate a cargo inside protein nanocages

Method	Nanocage	Cargo	Amount^a^	Ref.
pH	Ferritin	Doxorubicin (DOX)	30–90^b^	21 and 98–102
		Cisplatin	50, 11 to 51	103 and 104
		Quantum dots	1^c^	105
		Camptothecin	39	106
		Carboplatin	144	107
		Auoxo3, Auoxo4, Au2phen	Auoxo3 NR, Auoxo4 384, Au2phen 432	108
		Mitoxantrone	56 and 47	109
		Paclitaxel	60	110
		Gefitinib	∼10^d^	111
		siRNA	NR	112 and 113
	Encapsulin	CLP-functionalized AuNPs	9	114 and 115
	VLPs	siRNA	2–3 μM siRNAs	116
	Encapsulin	Gold nanoparticle (AuNP)	1	114 and 115

Chemical-based				
Urea	Ferritin	Epigallocatechin gallate (EGCG)	26.5	117
SDS	Ferritin	[Ru(bpy)_3_]^2+^ complex	4	118
Urea	VLP	Doxorubicin (DOX)	264 to 428	119
GuHCl	Encapsulin	Superfolder green fluorescent protein (sfGFP)	7	120
GuHCl	P22 VLP	Ferritin-scaffold fusion protein	1 to 5 ferritin nanocage	121 and 122
rMeTIR	Ferritin	Doxorubicin	NR	123
Hydrostatic pressure	Ferritin	None	NR	124

a Note: the amount is in number per cage unless otherwise stated.

b 404 DOX/nanocage (this is from DOI: 10.7150/thno.30867).

c Theoretically one QD/nanocage.

d Calculated based on the paper data.

These methods rely on breaking the interactions, including hydrophobic patches, hydrogen bonds, and salt bridges, at the interface of subunits under very harsh conditions to generate nanocage subunits and access the internal cavity. The subsequent removal of the chemical/physical force, in the presence of a stable drug, induces protein–protein interactions and spontaneous self-assembly, allowing the drug to be passively encapsulated. They are widely used to encapsulate cargo inside various natural protein nanocages. Among natural protein nanocages, the human H-type ferritin nanocage has garnered significant interest in the targeted delivery and imaging of cancer cells.^45,125–127^ This interest stems from the protein's interaction with the transferrin receptor, whose expression is highly induced in cancer cells due to their increased demand for iron.^128^ The capacity of ferritin to bind transferrin receptors is also associated with its ability to cross the blood–brain barrier and target brain tumours.^129,130^ Consequently, the use of ferritin nanocarriers in encapsulating hydrophilic anticancer drugs such as doxorubicin (DOX)^98^ or cisplatin^103^ has been extensively demonstrated (Table 1).

**Fig. 7:**
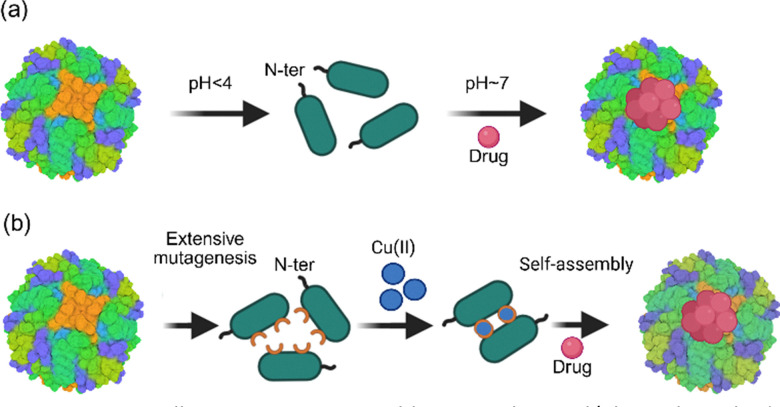
Controlling nanocage assembly using chemical/physical methods or extensive protein engineering. (a) The ferritin nanocage is disassembled under very acidic pH conditions and subsequently reassembled by adjusting the pH to neutral values. The addition of a drug to the disassembled nanocage at low pH results in drug encapsulation. (b) The reverse metal-templated interface redesign (rMeTIR) applied to control ferritin nanocage assembly. The method relies on extensive mutagenesis of the amino acid residues at the interface of two ferritin subunits to generate stable monomers and introduce Cu(ii) binding sites. The addition of Cu(ii) leads to the formation of dimers and subsequent self-assembly *via* a mechanism that is not fully understood.

The most widely used method for encapsulating a therapeutic, the pH-mediated disassembly/reassembly, involves changing the pH to highly acidic or basic values to disassemble nanocages. This step is followed by neutralisation in the presence of a small-molecule drug to reassemble the nanocages and encapsulate the drug (Fig. 7a). Engineering approaches are combined with physical and chemical methods to enhance the encapsulation of drugs. For example, engineering the C-terminus of each H-chain ferritin subunit with a peptide resulted in the formation of nanocages capable of simultaneously encapsulating both a hydrophobic and a hydrophilic drug.^106^ VLPs are engineered to create pH-responsive particles for targeted delivery.^131^ A common drawback of the chemical-physical methods of disassembly and reassembly is that they are pseudoreversible. The nanocages reassembled by changing the pH to neutral values partially lose their native structure, and/or some disassembled subunits are denatured and precipitate. Detailed studies using small-angle X-ray scattering (SAXS)^63^ revealed a stepwise disassembly process at a pH below 3 or 4. These studies showed that ferritin nanocages are partially reassembled when the pH is restored to 7.0.^63^ This partial reassembly can lead to drug leakage and toxicity. The pH-mediated drug encapsulation also leads to non-specific drug–protein interactions.

Removing these non-specific bindings requires additional chemical modification, such as cysteine–maleimide conjugation.^100^ While these physical/chemical methods cannot be easily applied to effectively encapsulate hydrophobic drugs, their use is also limited to stable drugs under harsh conditions, *e.g.* pH values well below or above the neutral pH. Furthermore, these methods cannot be easily combined with protein engineering approaches to decorate the nanocage's surface with a pH-sensitive protein, *e.g.*, viral antigen, and simultaneously encapsulate a drug to create multifunctional nanomedicines or nanocage vaccines that encapsulate widely used adjuncts such as aluminium hydroxide. Another bottleneck of physical or chemical methods for nanocage disassembly and reassembly to encapsulate a drug is scaling up the process to enable large-scale production and commercialisation. A fundamental challenge of scaling up a bioprocess is homogeneous mixing to ensure even reaction conditions within a few seconds.^132^ This is required to prevent localised extreme change in chemical properties, for example, a pH lower than 3–4, which will fully degrade ferritin. The damaged nanocages will not meet regulatory standards. This is an industrial limitation that has hindered the development and commercialisation of pH-mediated assembly or disassembly for creating a ferritin-based drug delivery platform, despite successes in laboratory-based animal studies.

### 4.2. Metal-induced interface assembly

Another approach to control ferritin nanocage assembly involved the mutagenesis of the interface between two ferritin subunits and the introduction of Cu(ii) binding sites. The reverse metal-templated interface redesign (rMeTIR) approach was introduced to disrupt the interaction of two ferritin subunits at the 2-fold symmetry axis.^62^ This approach required extensive mutagenesis to identify soluble subunits that could not spontaneously self-assemble into 24-meric nanocages. After obtaining soluble subunits, further mutagenesis was used to introduce two Cu(ii) binding sites at the interface between the two subunits (Fig. 7b). Consequently, subunits were assembled when Cu(ii) ions were added. The structural analyses using X-ray crystallography demonstrated the formation of 24-meric nanocages and the presence of two Cu(ii) ions at the interface between two subunits. The removal of Cu(ii) using a chelating agent led to the disassembly of nanocages and the formation of ferritin subunits. This approach was later used to encapsulate anticancer drug doxorubicin (DOX).^123^ Engineered subunits were mixed with a drug in the presence of Cu(ii) to induce self-assembly and passively encapsulate the cargo.

### 4.3. PINC technology

**Fig. 8:**
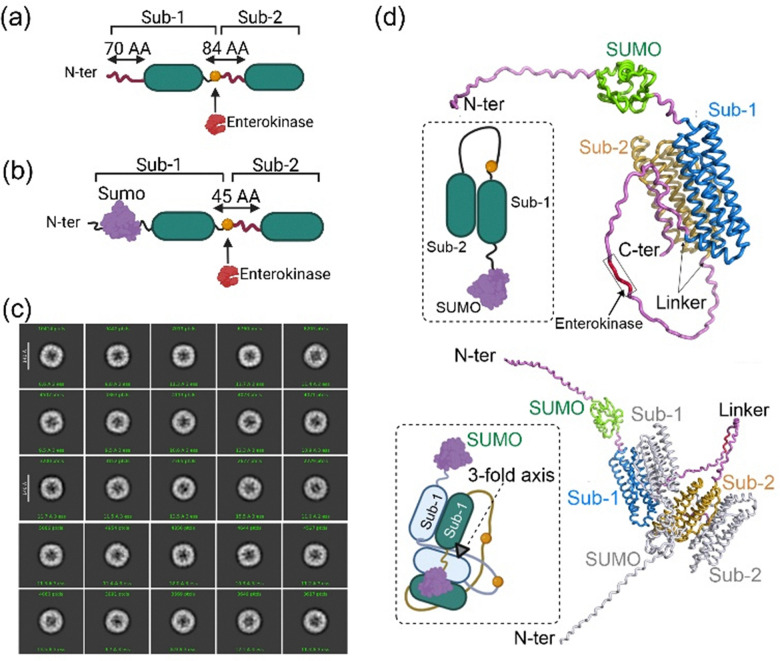
Development of PINC technology. (a) Simple engineering of ferritin subunits to create precursors of nanocages (PRECs). Each precursor is created by linking two ferritin subunits with a linker peptide, and (b) can be further modified by the addition of a protein to the N-terminus of a precursor. (c) 2D classification of the selected 24-meric PINC particles from cryo-EM micrographs. (d) The predicted structure of a PREC monomer or a PREC dimer. PREC monomers or dimers were observed using Native-PAGE and SEC. Reproduced from our work, ref. 39, permitted under CC-BY license.

To address the challenges associated with physical/chemical methods of controlling assembly and achieve concomitant drug encapsulation and surface functionalisation, we developed a new technology termed protease-induced nanocage (PINC) technology.^39^ We mimicked the HIV-1 capsid formation process, namely the conversion of Gag precursors to different protein domains by viral protease. As discussed earlier, the spontaneous assembly process of ferritin nanocage starts with the formation of dimers and their subsequent assembly. We hypothesised that if we attach the C-terminus of one ferritin subunit to the N-terminus of a second subunit *via* a flexible linker peptide with an enterokinase cleavage site, we will block self-assembly and enable assembly in the presence of a protease. To create a simple precursor of nanocages (PREC), two subunits of the hyperthermophilic ferritin from *Pyrococcus furiosus* were linked using a linker peptide encompassing the enterokinase cleavage site (Fig. 8a).

These precursors were overexpressed in *E. coli* and purified in a soluble form with a secondary and tertiary structure similar to that of the individual ferritin subunit, as observed by circular dichroism (CD) spectroscopy. Generating these precursors provides a platform for attaching different proteins or peptides to the N-terminus or C-terminus of ferritin subunits for surface functionalisation or encapsulation. This was demonstrated by fusing another protein, *e.g.*, the SUMO protein, to the N-terminus of the first subunit of precursors (Fig. 8b). Cleavage of these new precursors led to the formation of nanocages decorated with various numbers of SUMO proteins. A combination of Native-PAGE electrophoresis, size exclusion chromatography (SEC), and Cryo-EM revealed that upon addition of enterokinase, the precursors were converted to PINCs with a structure resembling native 24-meric ferritin nanocage (Fig. 8c). Native-PAGE and SEC analysis showed two states of precursors, monomers and dimers. Structural modelling using AlphaFold predicted that two precursors have the propensity to generate dimers of precursors, consisting of four ferritin subunits (Fig. 8d). These dimeric states partially form the 3-fold axis of the ferritin nanocage. However, because the two linker peptides are entangled, forming higher-order structures is impossible, and self-assembly is blocked. In making these dimers, the flexibility of the linker peptide is fundamental, as its replacement with a rigid α-helix peptide entirely abolished dimer formation.^39^ Interestingly, when dimers were not formed, the cleavage of precursors using enterokinase did not lead to the formation of 24-meric nanocages. Therefore, the linker peptide prevents the formation of hydrophobic patches, hydrogen bonds, and salt bridges between ferritin dimers, thereby blocking self-assembly. The addition of enterokinase cleaves the linker peptide, inducing conformational changes necessary for protein–protein interactions and nanocage assembly.

**Fig. 9:**
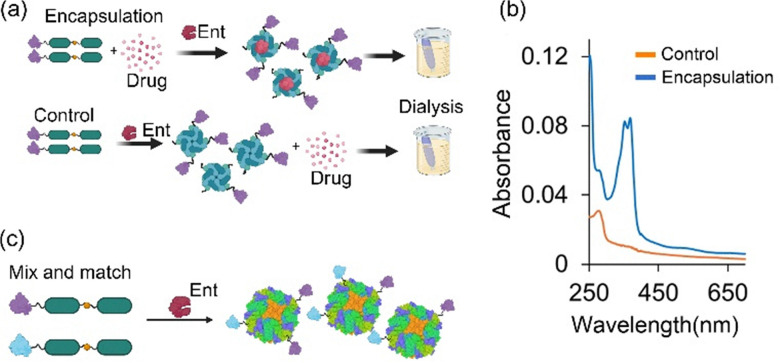
PINC technology capabilities to create multifunctional nanocages. (a) A simple one-step method can be used to encapsulate a drug inside nanocages and simultaneously decorate nanocages with a protein. This approach can be used to encapsulate hydrophilic drugs (*e.g.*, doxorubicin (DOX)) or (b) hydrophobic drugs (*e.g.*, camptothecin (CPT)). (c) PINC technology can be used to create mosaic nanocages decorated with at least two different proteins. Two different precursors are mixed, and after protease-cleavage assembly, they are matched, generating mosaic nanocages. Reproduced from our work, ref. 39, permitted under CC-BY license.

The PINC technology can be used in the presence of up to 30% DMSO, expanding the ferritin nanocage application to encapsulation of many poorly soluble and bioavailable drugs. Additionally, the technology enabled concomitant nanocage surface functionalisation and drug encapsulation. To determine if PINC technology can be used for the encapsulation of water-insoluble drugs (hydrophobic drugs), we first investigated whether PINCs were formed in the presence of various concentrations of DMSO. Precursors with an N-terminal SUMO protein were incubated in the presence of DMSO and enterokinase. The formation of PINCs was monitored using Native-PAGE. The results showed efficient PINC formation with minimum protein denaturation when the DMSO concentration was 30% or less (W/W). To establish the ability of PINC technology to efficiently encapsulate drugs, we developed a simple procedure (Fig. 9a). A drug, hydrophobic or hydrophilic, was added before PINC formation (encapsulation reaction) or after PINC formation (control). Subsequently, both the encapsulation reaction and the control free drug were subject to extensive dialysis to remove drug interactions with the nanocage surface and free drugs. Encapsulation of the drug was monitored using UV-visible spectrophotometry to monitor the amount of drug retained in nanocages after extensive dialysis. Comparing the encapsulation reaction with that of the control showed that both a hydrophilic (doxorubicin) and a hydrophobic (camptothecin) drug were efficiently encapsulated inside the nanocage, with no or negligible amount of drug tightly interacting with the nanocage surface (Fig. 9b). The amount of drug encapsulated was at least 2–3 times greater than that achieved using the widely applied pH-mediated disassembly/assembly method (Table 1). In PINC technology, the linker peptide between two subunits prevents self-assembly. In the presence of a drug, the addition of enterokinase cleaves the linker peptide, inducing conformational changes required for the formation of subunit interface interactions. Subsequent self-assembly leads to the passive encapsulation of a drug. Additionally, we demonstrated that different proteins could be added to the N-terminus and that different precursors could be mixed and matched after protease-cleavage assembly to generate mosaic nanocages, *i.e.*, nanocages decorated with more than one protein (Fig. 9c). These findings establish the PINC technology as a ‘plug and play’ platform for developing novel therapeutics.

## 5. Conclusion and perspectives

**Fig. 10:**
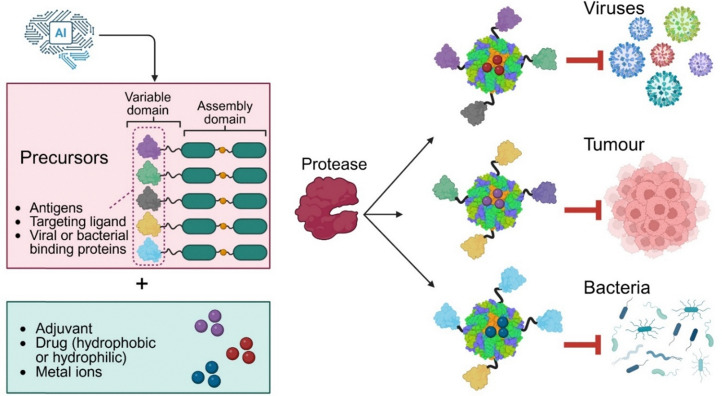
PINC Technology is a “plug and play” platform for sustainable manufacturing and development of various therapeutics. A machine-learning algorithm could be developed to rapidly design different variable domains and plug them into the assembly domain to create a library of precursors. The protease-cleavage assembly of these precursors, in the presence of adjuvants, drugs, or metal ions, will form therapeutics or prophylactics to target different entities, such as viruses, cancer cells, or bacteria.

Since the pioneering work by Robert Langer,^133–138^ the field of drug delivery has flourished with new synthetic^36,37^ or biobased technologies^30,31,139^ being developed to improve diagnosis, prevention, and therapy. Protein nanocages, such as ferritin, offer a unique platform for various applications in nanomedicine, including drug delivery and vaccine development.^140^ Animal and clinical trials have demonstrated the biocompatibility and safety of ferritin nanocages from different origins, including human, bacterial, and archaeal sources. The ferritin nanocage from the *H. pylori* bacterium or the hyperthermophilic archaeon *Pyrococcus furiosus*^141^ has been used to develop vaccine candidates and deliver drugs.^142–145^ By linking two ferritin subunits and creating precursors whose protease-cleavage self-assembly generates native-like 24-meric nanocages, we developed a safe and biocompatible “plug & play” platform for the development of various multifunctional nanomedicines. Each precursor is made of two domains: an assembly domain and a variable domain (Fig. 10). While the core assembly domain is fixed, the N-terminus or C-terminus can be used to add different therapeutic proteins or peptides using peptide plugs. Precursors can be mixed to construct multifunctional nanocages.

The cleavage-controlled assembly in the presence of a drug (both hydrophobic and hydrophilic) results in concomitant nanocage surface functionalisation and drug encapsulation. These unique properties offer applications in different areas. For example, precursors with varying antigens on their N-terminus could be mixed, and their protease-cleavage assembly could lead to the formation of mosaic vaccines while encapsulating an adjuvant such as aluminium oxide or an antiviral drug. This “plug and play” technology could be combined with an AI algorithm to rapidly design and optimise variable domains and modify the assembly domain for efficient protease-induced assembly and construction of nanocages with maximum therapeutic efficacy. This technology offers numerous advantages over other methods of controlling nanocage assembly (Table 2). However, there are challenges and potential limitations to using this technology for encapsulating drugs that act as protease inhibitors. These drugs might inhibit the protease cleavage-controlled assembly and nanocage formation. PINC technology, which utilises ferritin subunits, may not be suitable for delivering large proteins or RNA, as the nanocage's internal cavity has limited space.

**Table 2:** Unique selling point of PINC technology as compared to other ferritin nanocage assembly methods

Benefits	Attribute	PINC tech	Chemical/physical methods	rMeTIR
Assembly in the presence of a solvent	Both hydrophobic and hydrophilic drugs	✓	✗	✗
Lower cost and fewer steps	One-step construction^a^	✓	✗	✗
Broader application areas, faster and cheaper	Modular (plug and play)^b^	✓	✗	✗
No damage to protein	Compatible with regulatory needs & leakage-free^c^	✓	✗	✓
Benign conditions	Suitable for pH-sensitive drugs and ligands	✓	✗	✓

a Encapsulation and surface functionalisation can be simultaneously achieved in a single step.

b Different ligands can be mixed and matched on one nanocage, enabling construction of multifunctional therapeutics or preventives with application in different disease areas such as cancer and viral infection. It can be faster and cheaper to approve by mixing different already approved precursors and creating new therapeutic nanocages.

c Partial reassembly is reported using detailed SAXS analysis.^63^

The full realisation of this technology and its integration with AI requires further investigation to address several fundamental questions: What is the size limit of protein that can be added to the precursor N-terminus? How does the size and shape of the N-terminus protein affect the protease-cleavage and subsequent assembly of precursors? How does the sequence and size of the linker peptide between two subunits and the peptide plug between the N-terminus protein and the first subunit in the precursor affect cleavage-controlled assembly? Addressing these questions should help unfold the mechanism of nanocage assembly, further unleashing its potential for creating multifunctional nanomedicines.

Another question is the applicability of PINC technology to other protein nanocages. In the case of ferritin, the N- and C-termini of the subunits are not involved in protein–protein interactions, allowing for the modification of subunits and the creation of precursors. Therefore, extending PINC technology to other protein nanocages whose subunits’ C- or N-termini are involved in protein–protein interaction might prove challenging. We expect that the PINC technology can be extended to other protein nanocages, such as bacterioferritin, whose C- and N-termini are not involved in protein–protein interactions. We expect that the development and application of PINC technology lead to the generation and commercialization of multifunctional therapeutics and prophylactics such as mosaic vaccines mimicking a virus structure and at the same time encapsulating an adjuvant, targeted delivery of two or more therapeutic molecules, *e.g.* both hydrophobic and hydrophilic drugs, and nanocages deceiving a virus by presenting its cell-surface receptors^30^ and at the same time, encapsulating a drug to hijack the virus for targeted delivery at the time of viral entry.

## Author contributions

KHE conceived the idea; YS prepared all figures; YS and KHE wrote the initial draft of the manuscript; YS, MS, and KHE revised and finalized the manuscript.

## Conflicts of interest

There are no conflicts to declare.

## Data Availability

No new data were generated as part of this review.
